# Preoperative Peripheral Blood Serotonin and Kynurenine Levels Are Associated With Oncological Outcomes in Glioblastoma IDH-wt Patients

**DOI:** 10.1177/11786469241312475

**Published:** 2025-02-14

**Authors:** Silvia Snider, Filippo Gagliardi, Pierfrancesco De Domenico, Stefano Comai, Antonella Bertazzo, Sofia Nasini, Benedetta Barzon, Angela Ruban, Francesca Roncelli, Pietro Mortini

**Affiliations:** 1 Department of Neurosurgery and Gamma Knife Radiosurgery, IRCCS San Raffaele Scientific Institute, Milan, Italy; 2 Department of Pharmaceutical and Pharmacological Sciences, University of Padua, Italy; 3 Department of Biomedical Sciences, University of Padua, Italy; 4 Department of Psychiatry, McGill University, Montreal, QC, Canada; 5 Division of Neuroscience, IRCSS San Raffaele Scientific Institute, Milan, Italy; 6 Steyer School of Health Professions, Sackler Faculty of Medicine, Tel Aviv University, Israel

**Keywords:** Glioblastoma, kynurenine pathway, serotonin pathway, survival

## Abstract

**Background:**

In recent years, there has been a growing interest in exploring the potential contribution of tryptophan (TRP) metabolism via the kynurenine (KP) and serotonin (SP) pathways in Glioblastoma (GBM) biology. This study aims to address the association between pre-operative peripheral blood levels of TRP, kynurenine (KYN), 5-hydroxy-tryptophan (5-HTP), and serotonin (5-HT) and relevant oncological outcomes in GBM IDH-wt patients.

**Methods:**

This is a single-center, retrospective clinical study. Serum from 62 adult patients undergoing maximal safe resection of newly diagnosed glioblastoma WHO-grade 4 IDH-wt (GBM) and n = 27 healthy controls were analyzed. The variables of interest were dichotomized via maximally selected rank statistics. Kaplan Meier and Cox multivariate regression analysis were conducted to explore the single contributions of these parameters in building a predictive model of overall survival (OS) and progression-free survival (PFS) in these patients.

**Results:**

The mean baseline serum levels of 5-HT, KYN, and 5-HTP were significantly lower in GBM when compared to n = 27 healthy individuals (*P* < .001). Patients with 5-HT <78 ng/mL had a median OS of 14.4 months compared to 22.5 months in patients with increased levels (*P* = .01). Shorter OS was observed in patients with KYN <18 ng/mL (9.8 vs 17.5 months, *P* = .002), KYN/TRP <2.55 (11.4 vs 17.1, *P* = .002), 5-HTP/TRP <0.89 (11.5 vs 17.6 months, *P* = .02), and 5-HT/TRP <5.78 (13.4 vs 19.1 months, *P* = .002) compared to patients with high levels. Shorter PFS in patients with 5-HT <78 ng/mL (*P* = .04), KYN <18 ng/mL (*P* = .02), 5-HT/TRP <5.78 (*P* = .001), KYN/TRP <2.55 (*P* = .005). Reduced KYN, 5-HTP, and 5-HT were independent predictors of poor OS.

**Conclusions:**

This study highlights an intriguing association between the degradation of TRP along the KP and SP and median survival times in GBM. Decreased KYN, 5-HTP, and 5-HT levels were associated with shorter OS.

## Introduction

Glioblastoma (GBM) is the most common, fast-growing, and aggressive malignant primary CNS tumor, with a survival time of about 15 months.^1^ The incidence of GBM ranges from 3.19 cases to 4.17 per 100 000 person-years, with a peak incidence in people over 40 years of age.^2^ Current GBM treatment is multimodal and has not been substantially changed since 2005, despite remarkable efforts in neuro-oncological research. It consists of maximal safe resection surgery, followed by concurrent adjuvant radiotherapy and chemotherapy.^1^ Glioblastoma cells share with other malignant tumors the migratory capability, mainly through infiltration and invasion of surrounding brain parenchyma, thus making it difficult to distinguish the tumor cell mass from the surrounding healthy tissue. The understanding of specific molecular mechanisms underlying GBM growth is of strategic relevance to conceiving and developing new therapeutic approaches. Along the genetic background, the tumoral microenvironment plays a crucial role in sustaining GBM cells’ proliferation, energy supply, and immune escape. This microenvironment encompasses a set of cellular and molecular components that form the context in which the tumor initiates, proliferates, and eventually infiltrates surrounding normal tissue and actively participates in communication that ultimately supports tumor progression.^3^ Recent evidence suggests a pivotal role of tryptophan (TRP) metabolism via the kynurenine (KP) and serotonin (SP) pathways in this intricate peritumoral interplay.^4,5^

**Figure 1.**
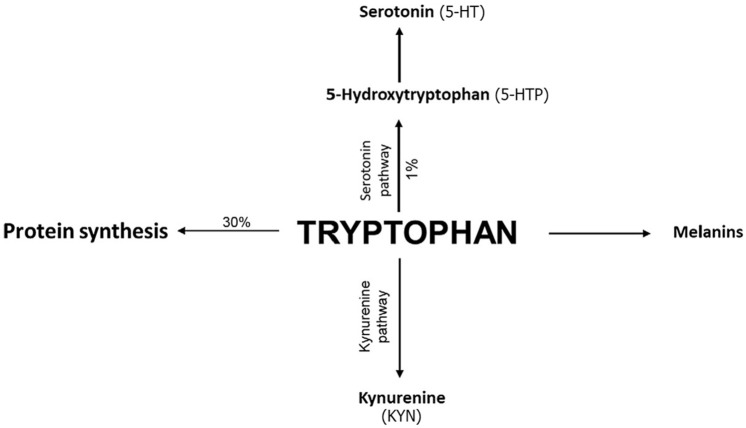
Tryptophan metabolism via the kynurenine and serotonin pathway. Adapted from Comai et al.^6^

The KP is the major route of TRP catabolism leading to the production of the essential pyridine nucleotide, nicotinamide adenine dinucleotide (NAD+), along with several downstream metabolites of kynurenine (KYN). Around 1% of TRP is used for the synthesis of serotonin (5-HT) along the SP,^6^ see Figure 1. The indoleamine 2,3-dioxygenase (IDO-1 and IDO-2) and tryptophan 2,3-dioxygenase (TDO-2) are the first and rate-limiting enzymes of the KP transforming TRP into formyl-KYN rapidly converted into KYN.^6^ Particularly, IDO-1 and TDO-2 expression is likely to play a role in mediating brain tumor immune evasion via inhibiting tumor-specific immunity and driving malignant progression.^7^ In physiological conditions, the peripheral KYN/TRP ratio mostly reflects TDO activity, while in inflammatory or tumoral conditions, IDO activity is likely to prevail.^6,8^ Hyperactivity of the KP may confer tumor survival advantages by increasing the availability of NAD+ for DNA repair, assisting in cell replication and maintenance of high metabolic activity, ultimately promoting tumor cell viability and proliferation.^9^ Moreover, the up-regulation of KP enzymes inhibits tumor-specific immunity through a combined effect of depletion of TRP from the tumor microenvironment, a reduction of cytotoxic T-cell infiltrates, and a significant increase in the immunosuppressant T-regulatory (T-reg) phenotype.^10^

The first step in the SP is the hydroxylation of TRP by the rate-limiting enzyme, tryptophan 5-hydroxylase (TPH), to 5-hydroxytryptophan (5-HTP). Changes in circulating TRP levels have been associated with an imbalance in its availability for the synthesis of 5-HTP and 5-HT in both the periphery and the central nervous system.^6,11^ 5-HT is known to mediate glioma cell growth and migration,^12,13^ while its role in neuroimmune circuits, interorgan communication, and inflammation and immunity associated with glioma is a new emerging research field. The exact mechanism and receptors promoting these events in glioma cells are still largely unknown.

The KP and SP metabolites can be easily measured in blood and might be promising hallmarks of TRP metabolic derangements in GBM. While the KYN/TRP plasma ratio is used as an indirect marker of KP pathway activation,^14-16^ the 5HTP/TRP and 5-HT/TRP plasma ratios might be a proxy of SP activity.^6,17^

## Materials and Methods

### Study design, patient selection, and data retrieval

This is a single-center, retrospective, observational clinical study designed to assess TRP metabolism via KP and SP in GBM patients. We included patients with adequate clinical follow-up who underwent maximal safe resection of newly diagnosed glioblastoma WHO grade 4 at IRCCS San Raffaele Hospital, Milan, Italy, between 2019 and 2022. Pediatric patients (<18 years old) and patients showing unresectable disease, undergoing biopsy only, IDH-mutated tumors, and recurrent GBM were excluded from the current analysis.

Diagnoses were originally performed according to the 2016 or 2021 WHO criteria, depending on the time of surgery. We only included “primary” (IDH 1-2 wild-type) GBM tumors. In detail, between 2019 and 2021, we only enrolled patients presenting a diagnosis of IDH-wt GBM through immunohistochemical detection of IDH1/IDH2 mutations. After 2021, only GBM IDH-wt, according to the latest WHO classification, was included. Pathological and molecular findings such as MGMT promoter methylation status, Ki-67 index, and p53 expression were reported. These parameters were included in the multivariate analysis to help identify the independent role of the study metabolites in predicting survival, given their well-known role in GBM. Indeed, MGMT promoter methylation is associated with better response to alkylating agents like temozolomide, the Ki-67 index reflects the proliferative activity of the tumor, and p53 expression is linked to tumor suppression and genomic stability.^18,19^

The TERT mutation was not routinely tested before 2021 in our center. Therefore, only a few patients had the mutation status revealed in this cohort.

Tumoral volumes were calculated on preoperative MRI imaging using cranial planning Brainlab software (Munich, Germany, 2021) on fluid-attenuated inversion recovery (FLAIR), T2-weighted (T2), and T1-weighted post-contrast sequences (T1-CE). The extent of resection (EOR) was calculated based on RANO criteria^20^ by 2 independent neurosurgeons on postoperative MRI scans performed for radiotherapy planning or immediate postoperative CT/MRI scans when available.

Baseline, adjuvant therapies, and follow-up clinical data were retrospectively retrieved from clinical records and included age, sex, and performance status.

Serum from n = 27 healthy volunteers was collected and analyzed as control samples. The control group had a higher female/male ratio (2 vs 0.55, *P* = .01) and younger age (42 ± 11 vs 64.2 ± 10, *P* < .001) compared to the GBM group.

### Blood sampling and processing

Preoperative peripheral blood samplings were routinely performed upon hospital admission, before any treatment, within 24 hours of surgery. Specimens were collected and stored in EDTA vacutainer venous blood collection tubes and separated by centrifugation (1500*g*, 15 minutes). The serum was then separated and stored at −80°C until the High-Performance Liquid Chromatography (HPLC) analysis was performed.

### Analysis of serum levels of TRP, KYN, 5-HTP, and 5-HT

According to a well-validated method in our laboratory,^21^ the separation and quantification of TRP, 5-HTP, and 5-HT were done with a Shimadzu LC-10AD HPLC system equipped with a Shimadzu RF-10AXL fluorometric detector set at excitation and emission wavelengths of 279 and 320 nm, respectively. Briefly, the chromatographic method consisted of an Apollo C18 (5 μm 250 mm × 4.6 mm) column (Sepachrom Mega Srl, Milan, Italy) and a mobile phase at a flow rate of 1 mL/min composed by 5% of a mix milliQ water/acetonitrile (5% water, 95% acetonitrile) and 95% of a mix milliQ water/methanol (90% water, 10% methanol) acidified with orthophosphoric acid to a pH of 3.5. KYN was quantified using the same mobile phase, a Robusta C18 (5 μm 250 mm × 4.6 mm) column (Sepachrom Mega Srl), and a Shimadzu SPD-10A UV–VIS detector set at 360 nm.

### Statistical analysis

Statistical analysis was conducted using R Core Team (2022), using survival (Therneau T, 2023), ggsurvfit (Sjoberg D, 2023), maxstat (Hothorn T, 2017), and ggplot2 (Wickham H, 2016) packages. Categorical variables are reported as absolute numbers and percentages, whereas continuous variables are reported as mean and standard deviation or median and interquartile range (IQR). The difference in baseline characteristics and the unadjusted univariate analyses were performed using the *t*-test or Mann-Whitney (*U*-test) in accordance with the normality of the distribution and Chi-square or Fisher’s exact test, where appropriate. Pearson’s correlation test was used to infer associations between demographics and serum biomarkers.

The continuous variables of interest (TRP, KYN, 5-HTP, 5-HT, 5-HT/TRP, KYN/TRP, 5-HTP/TRP) were dichotomized using the maximally selected rank statistics. The Kaplan-Meier method was used to estimate OS and PFS in the study population using the newly dichotomized variables. Log-rank tests analyzed differences between groups. Multivariate Cox regression analyses were used to detect variables associated with increased overall survival times. The results of all tests are presented as *P*-values, and statistical significance was set as a probability value of .05 (95% confidence interval).

## Results

### Patients and pathological characteristics

A total of 65 patients affected by IDH-wt GBM were originally screened. N = 3 cases with perioperative mortality (<1 month) were excluded: 2 suffered respiratory complications, one postoperative hemorrhage. Overall, 62 patients were included in the final analysis. The mean age was 64.2 ± 10.5 years. Most of the patients were male (n = 40, 65%) and aged <65 years old (n = 34, 54%). Globally, patients displayed a good functional status (KPS >80) in 60% of cases. Patients have been clinically followed up for a median of 13.7 months. See Table 1 for a summary of the baseline characteristics of included patients and lesions. Assessment of MGMT promoter status was available in 57 patients (92%) and revealed promoter hypermethylation in 39% of cases. The quantitative analysis of ki67 and p53 expression (positive cells) reported a mean of 28% ± 16 and 17% ± 22, respectively. The mean T1-CE volume was 28.1 cm^3^ ± 21.6. The extent of resection (EOR) was reported as complete or near total in 47 (76%), subtotal or partial in 15 (24%).

**Table 1.** Baseline characteristics of included patients and lesions characteristics.

Parameter	Included patients (n = 62)	Value	(%)
Epidemiology			
Demographics	Age	64.2 ± 10.5	—
	Male	40	65
	Female	22	35
Performance status	KPS >80	37	60
Lesion characteristics			
Location	Left hemisphere	32	51
	Frontal	22	35
	Temporal	35	56
	Insular	13	21
	Parietal	16	25
	Occipital	6	10
	Cerebellar	2	3
Radiology (MRI)	FLAIR (Vol, cm^3^)	95.6 ± 63.9	—
	T1-CE (Vol, cm^3^)	28.1 ± 21.6	—
	Necrosis (Vol, cm^3^)	8.9 ± 11	—
	Edema (Vol, cm^3^)	67.7 ± 50.4	—
	Central necrosis	54	86
	Midline shift	20	32
	Satellite FLAIR lesions	10	16
Molecular	MGMT (n = 57)	22	39
	ATRX loss (n = 60)	4	7
	KI67 (n = 62)	28% ± 16	—
	p53 (n = 61)	17% ± 22	—
EOR	Complete/near total	47	76
	Partial/subtotal	15	24
Adjuvant treatments	Treated in other centers	9	15
	Treated in our institution	53	85
	Radiation + chemotherapy protocol	51	82
	- RT full dose	34	64
	- RT short course	17	32
	- TMZ concurrent + adjuvant	38	72
	- Concurrent TMZ only	12	23
	- Experimental therapy	4	7
	TMZ only	3	6
Follow-up	Clinical follow-up	62	100
	Radiological follow-up	48	77
	Median clinical follow-up (m)	13.7 [9.2-20.6]	—
	Median radiological follow-up (m)	7.8 [6-12.6]	—
Outcomes	Median OS (m)	16.3 [13.4-21.7]	—
	Median PFS (m)	9.27 [7.77-13.9]	—

Abbreviations: EOR, extent of resection; Met, MGMT promoter methylation; TMZ, temozolomide; Vol, volume; m, months.

Values are expressed as numbers and percentages (%). Continuous variables are shown as median [interquartile range, IQR] or mean ± standard deviation, according to the normality of distribution.

Post-operative data on adjuvant radiotherapy (RT) and chemotherapy with temozolomide (TMZ) was available for the n = 53 (85%) patients treated in our Institutional radiotherapy and oncological services. Among them, n = 51 (94.6%) underwent radiation + chemotherapy protocol, n = 34 with full-dose (60 Gy, 30 fractions), and n = 17 with short-course (40 Gy, 15 fractions) RT regimens. N = 38 patients received concurrent + adjuvant TMZ regimen, while n = 12 only received the concurrent schedule without additional TMZ. Additional n = 4 patients received RT + experimental therapy.

### Baseline blood markers

Pearson correlation analyses revealed no association between age and TRP (*r* = .03, *P* = .77), KYN (*r* = −.17, *P* = .16), 5-HTP (*r* = .16, *P* = .21), and 5-HT (*r* = −.16, *P* = .20). Similarly, sex was not correlated to TRP (*r* = −.05, *P* = .69), KYN (*r* = −.007, *P* = .95), 5-HTP (*r* = −.14, *P* = .25, and 5-HT (*r* = .14, *P* = .25).

**Figure 2.**
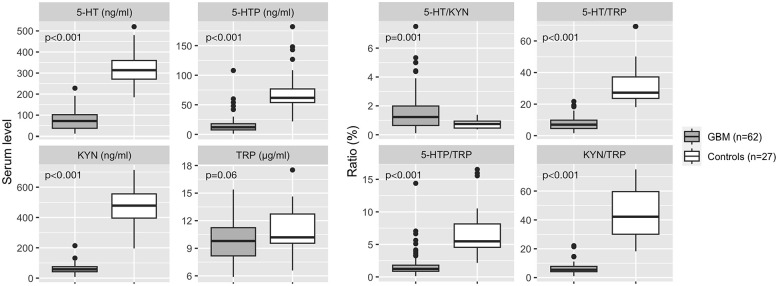
Comparison of GBM versus healthy controls baseline peripheral KP and SP metabolites. Boxplots represent median and interquartile ranges.

Mean baseline serum levels of 5-HT (76.9 ng/mL ± 45.9 vs 326.8 ng/mL ± 85.3, *P* < .001), KYN (61.2 ng/mL ± 33 vs 465 ng/mL ± 132.4, *P* < .001), KYN/TRP (6.40 ± 3.95 vs 44.9 ± 17.3, *P* < .001), 5-HT/TRP (7.86 ± 4.51 vs 31.2 ± 11.5, *P* < .001), and 5-HTP/TRP (1.93 ± 2.16 vs 6.99 ± 3.77, *P* < .001) were significantly lower in patients with GBM than in controls. A tendency for lower TRP levels in GBM than in controls (9.95 µg/mL ± 2.36 vs 10.9 µg/mL ± 2.51, *P* = .06) was also observed. See Table 2 and Figure 2.

**Table 2.** Baseline blood markers comparison between GBM and healthy controls.

Parameter	Variable	GBM (n = 62)	Controls (n = 27)	*P*-value
Demographics	Age	64.2 ± 10.05	42.07 ± 11.9	<.001
	Sex (M/F)	40/22	9/18	.01
Metabolites	TRP (µg/mL)	9.95 ± 2.36	10.9 ± 2.51	.06
	5-HTP (ng/mL)	17.6 ± 16.5	73.9 ± 37.2	<.001
	5-HT (ng/mL)	76.9 ± 45.9	326.8 ± 85.3	<.001
	KYN (ng/mL)	61.2 ± 33.01	465 ± 132.4	<.001
Ratio	5-HTP/TRP	1.93 ± 2.16	6.99 ± 3.77	<.001
	5-HT/TRP	7.86 ± 4.51	31.2 ± 11.5	<.001
	5-HT/KYN	1.71 ± 1.50	0.77 ± 0.30	.001
	KYN/TRP	6.40 ± 3.95	44.9 ± 17.3	<.001

Values are expressed as mean ± standard deviation.

### Overall survival (OS) analysis

#### Pre-operative laboratory parameters

The serum marker values were dichotomized using the maximally selected rank statistics for OS. The retrieved threshold for survival analysis was: TRP 10.6 µg/mL (*P* = 1.00); 5-HT 78 ng/mL (*P* = .14); KYN 18 ng/mL (*P* = .67); KYN/TRP <2.55 (*P* = 1.00); 5-HTP/TRP 0.89 (*P* = .43); 5-HT/TRP <5.78 (*P* = 1.00).

**Figure 3.**
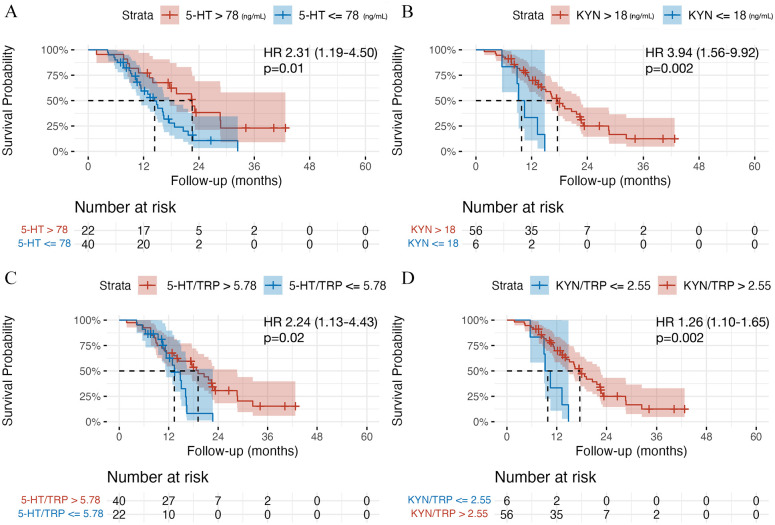
Kaplan-Meier curves for overall survival (OS) analysis. (A) 5-HT (ng/mL) levels high versus low. (B) KYN levels (ng/mL) high versus low. (C) 5-HT/TRP ratio high versus low. (D) KYN/TRP ratio high versus low. Abbreviation: HR, hazard ratio with 95% CI interval. *P*-values represent log-rank test results.

The median OS in the whole cohort of GBM patients was 16.3 months (95% CI: 13.4-21.7). Patients with 5-HT <78 ng/mL had a median OS of 14.4 months (95% CI: 11.4-17.6) compared to 22.5 months (95% CI: 17.7-27) in patients with 5-HT >78 ng/mL, *P* = .01. Shorter OS was recorded in patients with KYN <18 ng/mL (9.8 months, 95% CI: 11.4-17.6 vs 17.5 months, 95% CI: 14.4-22.5, *P* = .002), KYN/TRP <2.55 (11.4 months, 95% CI: 10.4-15.6 vs 17.1 months, 95% CI: 14.0-28.6, *P* = .002), 5-HTP/TRP <0.89 (11.5 months, 95% CI: 10.9-20.6 vs 17.6 months, 95% CI: 14.4-32.4, *P* = .02), and 5-HT/TRP <5.78 (13.4 months, 95% CI: 11.4-15.6 vs 19.1 months, 95% CI: 14.0-28.6, *P* = .02). See Figure 3.

#### Age, sex, performance status, MGMT methylation status

Patients older than 65 years had a median OS of 14.0 months (95% CI: 10.5-16.4), compared to 20.6 months (95% CI: 17.6-32.4, log-rank test *P* = .03) in younger patients. The median OS for the patients with low (<70) and high (⩾80) KPS were 16.1 (95% CI: 12.9-22.5) and 16.3 (95% CI: 10.5-22.4), respectively (log-rank test *P* = .90). Survival analysis for sex and MGMT methylation status did not reveal OS differences in our cohort of patients (*P* = .70 and *P* = .50, respectively), see Figure S1.

### Progression-free survival (PFS) analysis

**Figure 4.**
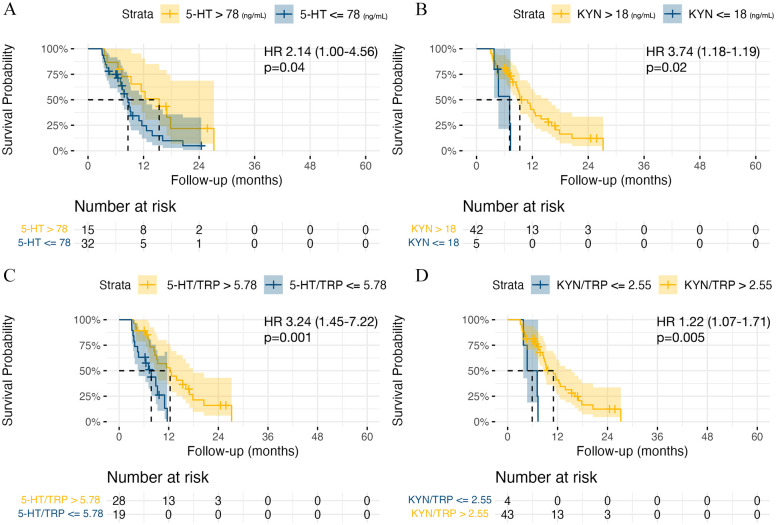
Kaplan-Meier curves for progression-free survival (PFS) analysis. (A) 5-HT levels high versus low. (B) KYN levels high versus low. (C) 5-HT/TRP ratio high versus low. (D) KYN/TRP ratio high versus low. Abbreviation: HR, hazard ratio with 95% CI interval. *P*-values represent log-rank test results.

The median PFS in the whole cohort was 16.3 months (95% CI: 13.4-21.7). Patients with 5-HT <78 ng/mL had a median PFS of 8.6 months (95% CI: 7.1-12.6) compared to 15.3 months (95% CI: 9.3-21.4) in patients with 5-HT >78 ng/mL, *P* = .04. Shorter PFS was detected in patients with KYN <18 ng/mL (7.1 months, 95% CI: 4.70-11.3 vs 9.3 months, 95% CI: 8.63-15.4, *P* = .02), KYN/TRP <2.55 (5.9 months, 95% CI: 3.77-9.8 vs 11 months, 95% CI: 8.63-15.4, *P* = .005), and 5-HT/TRP <5.78 (7.77 months, 95% CI: 4.70-10.4 vs 12.3 months, 95% CI: 8.63-17.8, *P* = .001), see Figure 4.

### Cox regression analysis

Multivariate Cox regression analysis of OS accounting for demographics, tumoral characteristics, EOR, and adjuvant therapies (*R*^2^ = 75%, *P* < .001) confirmed increased age (HR: 1.09, 95% CI: 1.02-1.15, *P* = .005), mean platelet volume (MPV, HR: 2.70, 95% CI: 1.40-5.22, *P* = .002), and Ki67 (HR: 62.9, 95% CI: 3.47-1142, *P* = .005) being associated with worse OS. Contrarily, increased baseline 5-HT (HR: 0.98, 95% CI: 0.97-0.99, *P* = .03), KYN (HR: 0.97, 95% CI: 0.94-0.99, *P* = .02), and 5-HTP (HR: 0.88, 95% CI: 0.81-0.96, *P* = .005) were independent predictors of increased OS. Increased 5-HT levels were also associated with longer PFS (HR: 0.97, 95% CI: 0.96-0.99, *P* = .001), together with increased 5-HTP (HR: 0.91, 95% CI: 0.85-0.97, *P* = .004). Increased MPV predicted worse PFS (HR: 2.69, 95% CI: 1.25-5.79, *P* = .001), Table 3.

**Table 3.** Multivariate Cox regression analysis of overall survival (OS) and progression-free survival (PFS) controlling for demographic and lesional parameters.

Parameter	Variable	Reference	OS			PFS		
			HR	95% CI	*P*-value	HR	95% CI	*P*-value
Demographic	Age	—	1.09	1.02-1.15	.005	1.01	0.93-1.09	.75
	Male	Female	1.51	0.44-5.12	.50	1.84	0.54-6.29	.32
	KPS <80	<80	0.60	0.21-1.70	.34	0.84	0.30-2.36	.74
Pathology	MGMT methy	Non methylated	0.63	0.20-1.98	.43	0.42	0.15-1.17	.09
	Ki67	—	62.9	3.47-1142	.005	0.11	0.001-8.0	.32
	p53	—	0.59	0.05-6.97	.68	2.11	0.19-23.2	.54
	Multifocal	Unifocal	3.30	0.55-19.6	.18	1.57	0.31-7.93	.58
EOR	Complete/near total	Subtotal/partial	1.11	0.35-3.48	.85	2.72	0.65-11.3	.16
Adjuvant therapies	Stupp protocol	TMZ only/none	0.13	0.03-0.52	.004	0.28	0.07-1.11	.07
Metabolites	5-HT (ng/mL)	—	0.98	0.97-0.99	.03	0.97	0.96-0.99	.001
	KYN (ng/mL)	—	0.97	0.94-0.99	.02	1.00	0.98-1.02	.74
	5-HTP (ng/mL)	—	0.88	0.81-0.96	.005	0.91	0.85-0.97	.004
	MPV	—	2.70	1.40-5.22	.002	2.69	1.25-5.79	.001

Abbreviation: KPS, Karnofsky performance status.

## Discussion

This study revealed that TRP metabolism is significantly altered in GBM patients, as reflected in a reduction of serum levels of KYN, 5-HTP, and 5-HT compared with healthy controls. Greater decreases are associated with worse OS and are independent risk factors for decreased survival. Additionally, lower 5-HTP and 5-HT are associated with decreased PFS.

### Tryptophan metabolism via the Kynurenine pathway in GBM

The KP has received increasing attention in cancer pathology due to its implication in tumor progression and immune modulation.^22,23^ Its involvement has been described in different tumors, including primary brain, breast, lung, colon, and renal cancer. The induction of the rate-limiting enzymes initiating the KYN pathway (IDO-1/2 and TDO-2) is the key aspect of KP activation in GBM. Tumoral cells express IDO1 due to the loss of tumor-suppressor gene Bin1^14^ or when identified by tumor-infiltrating immune cells and exposed to pro-inflammatory cytokine IFN-γ.^24,25^ Concerning other enzymes of the KP, it has been shown that the kynurenine aminotransferases (KAT) are downregulated,^4^ while the quinolinate phosphoribosyl-transferase (QPRT) that catalyzes the conversion of quinolinic acid (QA) to nicotinic acid mononucleotide, the final step in the biosynthesis of NAD+ coenzyme, is hyperactive, thus conferring tumor survival advantages, assisting in cell replication, and maintaining high metabolic activity.^9^ The up-regulation of key KP enzymes has been observed in other cells comprising the tumoral microenvironment (particularly the tumor-infiltrating macrophages and other antigen-presenting cells) and is known to inhibit tumor-specific immunity through a combined effect of depletion of TRP^26,27^ and direct activity of KP intermediates (ie, KYN, 3-HAA, and 3-HK) that are associated with a reduction of CD8+ cytotoxic T-cells infiltrate and a significant increase in the immunosuppressant CD4+/CD25+/FOXP3+ (Tregs) phenotype.^10^ Particularly, TRP starvation is known to promote T-cell autophagy and anergy,^27^ while KYN potently binds to cytoplasmic aryl hydrocarbon receptor (AhR), which promotes a crude reduction of CD8+ T-cell infiltration.^28,29^ All these elements contribute to the peri- and intra-tumoral accumulation of immunosuppressive T-regs that may worsen GBM overall prognosis.^10,30^ Higher KP activity is also associated with resistance to treatments such as immune checkpoint inhibitors (anti-PD-1), ipilimumab (anti-CTLA-4), NAD+ inhibitors, and chemotherapeutic agents such as cisplatin and paclitaxel.^31,32^

To sum up, KP hyperactivity is thought to promote tumoral persistence by skewing the KP away from the neuroprotective branches of the pathway, increasing the tumor’s ability to produce NAD+ to support cellular metabolism and proliferation, and mediating brain tumor immune evasion and resistance to therapies. Over-activation of KP is known to increase the consumption of TRP by glioma cells and a substantial modification in downstream KP substrate concentrations within cells and the peritumoral micro-environment. The final changes in local concentrations of substrates are partially reflected in peripheral blood through the brain-blood barrier. Only a few authors have analyzed the peripheral levels of KP substrates in GBM models and patients and found the KYN/TRP ratio as a promising biomarker for predicting the clinicopathologic status of tumors to GBM progression. Adams et al^4^ characterized the KP in plasma from GBM patients (n = 18) and demonstrated an overall reduction of TRP (−61%) and KYN (−37%) when compared to healthy individuals. They found increased KYN/TRP (+188%) in their cohort of patients due to the more consistent decrease in TRP in relation to KYN. Little information was given regarding the nature of controls and the timing of sampling, and the study analyzed a non-homogeneous cohort of GBM (naïve and recurrent), IDH-mutated and -wt, and other neuronal/glial malignancies. A recent report by Zhai et al^33^ reported a significant reduction in serum KYN levels (−70%) and a slightly smaller decrease in TRP levels (−68%), resulting in decreased KYN/TRP ratio (−7%) in a small cohort series of GBM patients (n = 10) compared to controls. Interestingly, KYN and TRP levels returned to values comparable to those of controls 10 weeks post-surgery. Following the postoperative surge, an elevated KYN/TRP ratio was found to be predictive of poorer OS. The authors suggested that the late increase in the KYN/TRP ratio could be linked to the induction of IDO-1/2 or TDO2 by treatments such as chemo/radiotherapy and/or dexamethasone or to systemic IDO-1 induction associated with immune activation upon recurrence. Accordingly, Panitz et al,^34^ analyzing a cohort of 43 recurrent GBM patients, observed a slightly more pronounced reduction in serum TRP levels (−18%) relative to KYN (−14%) compared to controls. They also noted that the magnitude of reduction decreased with the number of enzymatic steps between TRP and its metabolites, with the smallest reduction observed for downstream products. The authors proposed that this could be related to the overall TRP availability, which controls the levels of its systemic metabolites. In our study, which includes the largest homogeneous cohort of naïve-treatment IDH-wt patients, we observed a trend for TRP reduction (−10%) accompanied by a pronounced KYN depletion (−87%) and a corresponding decrease in the KYN/TRP ratio (−86%), before any treatment. This substantial reduction in KYN levels and the relatively lower KYN/TRP ratio in treatment-naive GBM aligns with the findings by Zhai et al^33^ before disease recurrence. This data suggests that the extent of TRP metabolism dysregulation may vary depending on the stage of the disease and the treatments being administered, such as chemotherapy or radiotherapy. Notably, we found that a greater reduction in KYN levels before treatment was associated with poorer prognosis, underscoring the central role of TRP metabolism in GBM. Downstream of KYN, Adams et al^4^ observed that both the neuroprotective compounds kynurenic acid (KYNA) and picolinic acid (PIC) levels were decreased in the serum of GBM patients, while Kesarwani et al^35^ observed that quinolinic acid (QUIN) accumulates in GBM and contributes to immune tolerance state by polarizing macrophage toward an anti-inflammatory and tumor-supportive phenotype (M2-like).

Collectively, these data suggest that pre-operative KYN and TRP levels potentially reflect the extent of KP dysregulation in GBM and might exert a prognostic role in predicting improved survival times for patients offered surgery and adjuvant treatments. KP dysregulation has received increasing attention in cancer GBM and is under active investigation to develop molecules aiming to modulate its activity. Our study represents the largest cohort (n = 62) supporting the involvement of KP in GBM pathology and the ongoing efforts to target this pathway as a potential therapeutic approach.

Indeed, Preclinical models have shown that inhibiting the KP can attenuate GBM progressions. IDO1 inhibitors, such as INCB024360, have garnered increasing attention,^36^ and subsequent studies have demonstrated that combining IDO1 inhibition with immune checkpoint inhibitors could significantly enhance therapeutic efficacy.^37^ One study found that dual inhibition of IDO-1 and TDO with PVZB3001 suppressed tumoral growth and decreased the KYN/TRP ratio.^38^ Building on these promising preclinical findings, clinical trials are now investigating the efficacy of IDO-1 inhibitors in GBM. Several IDO1 enzyme inhibitors or KP modulators are currently being evaluated in clinical trials (for a detailed review, see Tang et al^39^). Notable trials include Epacadostat in conjunction with radio/chemotherapy and bevacizumab (NCT03532295), BMS-986205 with nivolumab/radiotherapy or chemoradiotherapy (NCT04047706), and indoximod combined with TMZ, radiation therapy (NCT025027), and bevacizumab (NCT02052), which are currently recruiting recurrent GBM patients. Interestingly, in a phase 1 trial, the IDO-1 inhibitor PF-06840003 demonstrated disease control in 47% of patients (n = 8).^40^ These promising results highlight the importance of further investigating KP dysregulation in GBM patients and suggest that evaluating TRP metabolites could aid in the identification of patient cohorts most likely to benefit from targeted interventions aimed at these pathways.

### Tryptophan metabolism via Serotonin pathway in GBM

The SP, the other major metabolic pathway of TRP, has been far less investigated in GBM pathology compared to the KP. In this work, we observed significantly reduced systemic 5-HT levels in treatment-naïve GBM patients. To date, no other studies have addressed the peripheral levels of 5-HT in GBM. The critical step in 5-HT synthesis is highly dependent on TRP availability, and changes in TRP are known to impact both the systemic and brain 5-HT production.^11,14,29^ Thus, it can be hypothesized that the observed reduction in TRP levels could influence the overall 5-HT synthesis.

The exact role of 5-HT in GBM remains unexplored for several aspects.^8^ 5-HT has been implicated in the modulation of several cancer-related pathways, including PI3K/AKT and MAPK, which are critical for cell survival and proliferation.^41^ Moreover, various 5-HT receptors are expressed on GBM cells, suggesting that they may influence tumor behavior via receptor-mediated mechanisms. However, only a limited number of studies have examined the expression of 5-HT receptors in GBM models. For example, the 5-HTR5A was found to be downregulated in GBM compared to low-grade gliomas, and its stimulation enhanced GBM autophagy and apoptosis.^22^ Conversely, the 5-HTR7A was identified as promoting IL-6 release by U373 GBM cells, thereby facilitating tumoral progression via an autocrine pathway.^23^ Additionally, 5-HT may modulate the tumor microenvironment, potentially influencing the immune response to tumors. 5-HT has been shown to affect the behavior of macrophages and T-cells, which play critical roles in tumor immune surveillance. Although 5-HT signaling has been explored in T cells, the precise mechanism by which it influences T cell differentiation and functions remains unclear. Given that the specific pattern of 5-HT receptor expression involved in GBM progression has not been fully characterized, the extent and nature of SP dysregulation in GBM remain uncertain.

In this study, reduced systemic 5-HT levels were associated with poorer OS despite standard-of-care treatments. This result might be explained by the specific pattern of 5-HT receptors in GBM cells, which could promote a more aggressive course of the disease when systemic 5-HT levels are reduced. Alternatively, systemic platelet activation could account for this reduction. Peripheral 5-HT is primarily synthesized in enterochromaffin cells, released into the bloodstream, and absorbed by circulating platelets, forming a potent 5-HT reservoir. In the thrombotic milieu of tumors, platelet aggregation frequently occurs, leading to the release of large amounts of 5-HT, which may result in higher local concentrations than those in the bloodstream. Interestingly, systemic platelet activation has been linked to GBM progression and angiogenesis.^42,43^ In this study, elevated MPV further confirmed its independent predictive role for poorer OS. In summary, 5-HT appears to play a multifaceted role in GBM biology, influencing tumor growth in the tumor microenvironment and potentially offering new avenues for therapeutic intervention. Most clinical studies on 5-HT and cancer have focused on the relationship between tumors and depressive disorders, as well as the use of antidepressants. Depression and psychological distress are common in cancer patients,^44^ and in those with primary malignant brain tumors, it has been associated with a poorer OS.^45,46^ This has led to the hypothesis that selective serotonin reuptake inhibitor (SSRI) might positively affect the survival of patients with GBM. Caudill et al^47^ reported a survival benefit with the use of SSRIs, though this finding was not replicated in a larger retrospective study (n = 497).^48^ These data raise the question of whether the negative impact of low serum levels of 5-HT on OS and PFS in GBM is an indirect effect of metabolic imbalance toward KP, a direct consequence of systemic depletion of 5-HT, possibly including its downstream metabolite melatonin, or both. Additional studies are necessary to clarify the role of TRP metabolism in GBM. The lack of clarity regarding which 5-HT receptors are involved complicates our understanding of how 5-HT influences tumor behavior, whether it promotes or inhibits growth, and how it might be leveraged therapeutically. Further research is necessary to delineate these pathways and determine how they could be exploited in treating glioblastoma.

### Limitations

The main limitations of this study include the monocentric design, the relatively small sample size, the absence of comprehensive molecular analysis (eg, full MGMT methylation profile, TERT, and EGFR mutations), and the lack of assessment of downstream metabolites of KYN. The study did not include a longitudinal analysis of the biomarkers following surgery and treatment and did not provide a sex-matched control cohort.

## Conclusions

This study unveils an intriguing connection between KP activation, TRP consumption, and a relative reduction in 5-HT synthesis, potentially providing GBM cells with a survival advantage. Additional studies are needed to further analyze this interrelation and to identify the individual contributions of KP, SP, platelets activation, and immune infiltrate in the progression of GBM. In particular, the analysis of downstream metabolites of the KP will help to have a better biological framework for linking TRP metabolism to GBM. This approach could lay the groundwork for exploring the therapeutic potential of interventions targeting these systems, thereby enhancing therapeutic approaches for GBM patients.

## Supplemental Material

sj-tif-1-try-10.1177_11786469241312475 – Supplemental material for Preoperative Peripheral Blood Serotonin and Kynurenine Levels Are Associated With Oncological Outcomes in Glioblastoma IDH-wt Patients Supplemental material, sj-tif-1-try-10.1177_11786469241312475 for Preoperative Peripheral Blood Serotonin and Kynurenine Levels Are Associated With Oncological Outcomes in Glioblastoma IDH-wt Patients by Silvia Snider, Filippo Gagliardi, Pierfrancesco De Domenico, Stefano Comai, Antonella Bertazzo, Sofia Nasini, Benedetta Barzon, Angela Ruban, Francesca Roncelli and Pietro Mortini in International Journal of Tryptophan Research

## References

[1] Stupp R Mason WP Van Den Bent MJ , et al. Radiotherapy plus concomitant and adjuvant temozolomide for glioblastoma. N Engl J Med. 2005;352:987-996. 15758009 10.1056/NEJMoa043330

[2] Fabbro-Peray P Zouaoui S Darlix A , et al. Association of patterns of care, prognostic factors, and use of radiotherapy-temozolomide therapy with survival in patients with newly diagnosed glioblastoma: a French national population-based study. J Neurooncol. 2019;142:91-101. 30523606 10.1007/s11060-018-03065-z PMC6399437

[3] Sharma P Aaroe A Liang J Puduvalli VK. Tumor microenvironment in glioblastoma: current and emerging concepts. Neurooncol Adv. 2023;5:vdad009. 10.1093/noajnl/vdad009 PMC10034917 36968288

[4] Adams S Teo C McDonald KL , et al. Involvement of the kynurenine pathway in human glioma pathophysiology. PLoS One. 2014;9:e112945. 10.1371/journal.pone.0112945 PMC4240539 25415278

[5] Jacquerie A Hoeben A Eekers DB , et al. High expression of kynurenine pathway markers in glioblastoma: prognostic relevance. Preprint. Posted online June 27, 2024. doi:10.21203/rs.3.rs-4112388/v1 PMC11217262 38951170

[6] Comai S Bertazzo A Brughera M Crotti S. Tryptophan in health and disease. Adv Clin Chem. 2020;95:165-218. 32122523 10.1016/bs.acc.2019.08.005

[7] Zhai L Bell A Ladomersky E , et al. Immunosuppressive IDO in cancer: mechanisms of action, animal models, and targeting strategies. Front Immunol. 2020;11:1185. 32612606 10.3389/fimmu.2020.01185 PMC7308527

[8] Schrocksnadel K Wirleitner B Winkler C Fuchs D. Monitoring tryptophan metabolism in chronic immune activation. Clin Chim Acta. 2006;364:82-90. 16139256 10.1016/j.cca.2005.06.013

[9] Adams S Braidy N Bessesde A , et al. The kynurenine pathway in brain tumor pathogenesis. Cancer Res. 2012;72:5649-5657. 23144293 10.1158/0008-5472.CAN-12-0549

[10] Wainwright DA Balyasnikova IV Chang AL , et al. IDO expression in brain tumors increases the recruitment of regulatory T cells and negatively impacts survival. Clin Cancer Res. 2012;18:6110-6121. 22932670 10.1158/1078-0432.CCR-12-2130 PMC3500434

[11] Fernstrom JD Wurtman RJ. Brain serotonin content: increase following ingestion of carbohydrate diet. Science. 1971;174:1023-1025. 5120086 10.1126/science.174.4013.1023

[12] Merzak A Koochekpour S Fillion MP Fillion G Pilkington GJ. Expression of serotonin receptors in human fetal astrocytes and glioma cell lines: a possible role in glioma cell proliferation and migration. Brain Res Mol Brain Res. 1996;41:1-7. 8883928 10.1016/0169-328x(96)00058-7

[13] Sarrouilhe D Mesnil M. Serotonin and human cancer: a critical view. Biochimie. 2019;161:46-50. 29936294 10.1016/j.biochi.2018.06.016

[14] Comai S Bertazzo A Vachon J , et al. Tryptophan via serotonin/kynurenine pathways abnormalities in a large cohort of aggressive inmates: markers for aggression. Prog Neuropsychopharmacol Biol Psychiatry. 2016;70:8-16. 27117820 10.1016/j.pnpbp.2016.04.012

[bibr15-11786469241312475] Tong Q Song J Yang G Fan L Xiong W Fang J. Simultaneous determination of tryptophan, kynurenine, kynurenic acid, xanthurenic acid and 5-hydroxytryptamine in human plasma by LC-MS/MS and its application to acute myocardial infarction monitoring. Biomed Chromatogr. 2018;32:e4156. 10.1002/bmc.4156 29193181

[16] Badawy AA Guillemin G. The plasma [kynurenine]/[tryptophan] ratio and indoleamine 2,3-dioxygenase: time for appraisal. Int J Tryptophan Res. 2019;12:1178646919868978. 31488951 10.1177/1178646919868978 PMC6710706

[17] Yao JK Dougherty GG Reddy RD , et al. Altered interactions of tryptophan metabolites in first-episode neuroleptic-naive patients with schizophrenia. Mol Psychiatry. 2010;15:938-953. 19401681 10.1038/mp.2009.33 PMC2953575

[18] Hegi ME Diserens AC Gorlia T , et al. MGMT gene silencing and benefit from temozolomide in glioblastoma. N Engl J Med. 2005;352:997-1003. 15758010 10.1056/NEJMoa043331

[19] Louis DN Perry A Reifenberger G , et al. The 2016 World Health Organization classification of tumors of the central nervous system: a summary. Acta Neuropathol. 2016;131:803-820. 27157931 10.1007/s00401-016-1545-1

[20] Karschnia P Young JS Dono A , et al. Prognostic validation of a new classification system for extent of resection in glioblastoma: a report of the RANO resect group. Neuro Oncol. 2023;25:940-954. 35961053 10.1093/neuonc/noac193 PMC10158281

[21] Inserra A Giorgini G Lacroix S , et al. Effects of repeated lysergic acid diethylamide (LSD) on the mouse brain endocannabinoidome and gut microbiome. Br J Pharmacol. 2023;80:721-739. 10.1111/bph.15977 36316276

[22] Platten M Nollen EA Röhrig UF Fallarino F Opitz CA. Tryptophan metabolism as a common therapeutic target in cancer, neurodegeneration and beyond. Nat Rev Drug Discov. 2019;18:379-401. 30760888 10.1038/s41573-019-0016-5

[23] Girithar HN Staats Pires A Ahn SB Guillemin GJ Gluch L Heng B. Involvement of the kynurenine pathway in breast cancer: updates on clinical research and trials. Br J Cancer. 2023;129:185-203. 37041200 10.1038/s41416-023-02245-7 PMC10338682

[24] Zhai L Ladomersky E Lauing KL , et al. Infiltrating T cells increase IDO1 expression in glioblastoma and contribute to decreased patient survival. Clin Cancer Res. 2017;23:6650-6660. 28751450 10.1158/1078-0432.CCR-17-0120 PMC5850948

[25] Werner-Felmayer G Werner ER Fuchs D Hausen A Reibnegger G Wachter H. Neopterin formation and tryptophan degradation by a human myelomonocytic cell line (THP-1) upon cytokine treatment. Cancer Res. 1990;50:2863-2867. 2110500

[26] Godin-Ethier J Hanafi LA Piccirillo CA Lapointe R. Indoleamine 2,3-dioxygenase expression in human cancers: clinical and immunologic perspectives. Clin Cancer Res. 2011;17:6985-6991. 22068654 10.1158/1078-0432.CCR-11-1331

[27] Liu M Wang X Wang L , et al. Targeting the IDO1 pathway in cancer: from bench to bedside. J Hematol Oncol. 2018;11:100. 30068361 10.1186/s13045-018-0644-y PMC6090955

[28] Opitz CA Litzenburger UM Sahm F , et al. An endogenous tumour-promoting ligand of the human aryl hydrocarbon receptor. Nature. 2011;478:197-203. 21976023 10.1038/nature10491

[29] Obara-Michlewska M. The tryptophan metabolism, kynurenine pathway and oxidative stress - implications for glioma pathobiology. Neurochem Int. 2022;158:105363. 35667490 10.1016/j.neuint.2022.105363

[30] Facciabene A Motz GT Coukos G. T-regulatory cells: key players in tumor immune escape and angiogenesis. Cancer Res. 2012;72:2162-2171. 22549946 10.1158/0008-5472.CAN-11-3687 PMC3342842

[31] Meireson A Devos M Brochez L. IDO expression in cancer: different compartment, different functionality? Front Immunol. 2020;11:531491. 33072086 10.3389/fimmu.2020.531491 PMC7541907

[32] Botticelli A Cerbelli B Lionetto L , et al. Can IDO activity predict primary resistance to anti-PD-1 treatment in NSCLC? J Transl Med. 2018;16:219. 30081936 10.1186/s12967-018-1595-3 PMC6080500

[33] Zhai L Dey M Lauing KL , et al. The kynurenine to tryptophan ratio as a prognostic tool for glioblastoma patients enrolling in immunotherapy. J Clin Neurosci. 2015;22:1964-1968. 26279502 10.1016/j.jocn.2015.06.018 PMC4548799

[34] Panitz V Končarević S Sadik A , et al. Tryptophan metabolism is inversely regulated in the tumor and blood of patients with glioblastoma. Theranostics. 2021;11:9217-9233. 34646367 10.7150/thno.60679 PMC8490504

[35] Kesarwani P Kant S Zhao Y , et al. Quinolinate promotes macrophage-induced immune tolerance in glioblastoma through the NMDAR/PPARgamma signaling axis. Nat Commun. 2023;14:1459. 36927729 10.1038/s41467-023-37170-z PMC10020159

[36] Sordillo PP Sordillo LA Helson L. The kynurenine pathway: a primary resistance mechanism in patients with glioblastoma. Anticancer Res. 2017;37:2159-2171. 28476779 10.21873/anticanres.11551

[37] Ladomersky E Zhai L Lenzen A , et al. IDO1 inhibition synergizes with radiation and PD-1 blockade to durably increase survival against advanced glioblastoma. Clin Cancer Res. 2018;24:2559-2573. 29500275 10.1158/1078-0432.CCR-17-3573 PMC5984675

[38] Yoshioka S Ikeda T Fukuchi S , et al. Identification and characterization of a novel dual inhibitor of indoleamine 2,3-dioxygenase 1 and tryptophan 2,3-dioxygenase. Int J Tryptophan Res. 2022;15:11786469221138456. 36467776 10.1177/11786469221138456 PMC9716449

[39] Tang K Wu YH Song Y Yu B. Indoleamine 2,3-dioxygenase 1 (IDO1) inhibitors in clinical trials for cancer immunotherapy. J Hematol Oncol. 2021;14:68. 33883013 10.1186/s13045-021-01080-8 PMC8061021

[40] Reardon DA Desjardins A Rixe O , et al. A phase 1 study of PF-06840003, an oral indoleamine 2,3-dioxygenase 1 (IDO1) inhibitor in patients with recurrent malignant glioma. Invest New Drugs. 2020;38:1784-1795. 32436060 10.1007/s10637-020-00950-1

[41] Park S Kim Y Lee J , et al. A systems biology approach to investigating the interaction between serotonin synthesis by tryptophan hydroxylase and the metabolic homeostasis. Int J Mol Sci. 2021;22:2452. 33671067 10.3390/ijms22052452 PMC7957782

[42] Wach J Apallas S Schneider M , et al. Mean platelet volume/platelet count ratio and risk of progression in glioblastoma. Front Oncol. 2021;11:695316. 34178693 10.3389/fonc.2021.695316 PMC8221069

[43] Alimohammadi E Bagheri SR Bostani A Rezaie Z Farid M. Preoperative platelet distribution width-to-platelet count ratio as a prognostic factor in patients with glioblastoma multiforme. Br J Neurosurg. 2024;38:307-313. 33356619 10.1080/02688697.2020.1864293

[44] Hartung TJ Friedrich M Johansen C , et al. The Hospital Anxiety and Depression Scale (HADS) and the 9-item Patient Health Questionnaire (PHQ-9) as screening instruments for depression in patients with cancer. Cancer. 2017;123:4236-4243. 28654189 10.1002/cncr.30846

[45] Shi C Lamba N Zheng LJ , et al. Depression and survival of glioma patients: a systematic review and meta-analysis. Clin Neurol Neurosurg. 2018;172:8-19. 29957299 10.1016/j.clineuro.2018.06.016

[46] Gathinji M McGirt MJ Attenello FJ , et al. Association of preoperative depression and survival after resection of malignant brain astrocytoma. Surg Neurol. 2009;71:299-303, discussion 303. 18786716 10.1016/j.surneu.2008.07.016

[47] Caudill JS Brown PD Cerhan JH Rummans TA. Selective serotonin reuptake inhibitors, glioblastoma multiforme, and impact on toxicities and overall survival: the mayo clinic experience. Am J Clin Oncol. 2011;34:385-387. 20859197 10.1097/COC.0b013e3181e8461a

[48] Otto-Meyer S DeFaccio R Dussold C , et al. A retrospective survival analysis of Glioblastoma patients treated with selective serotonin reuptake inhibitors. Brain Behav Immun Health. 2020;2:100025. 32190845 10.1016/j.bbih.2019.100025 PMC7079579

