# Supplementary figures and images for: Preoperative Peripheral Blood Serotonin and Kynurenine Levels Are Associated With Oncological Outcomes in Glioblastoma IDH-wt Patients
Source: Int J Tryptophan Res. 2025 Feb 14;18:11786469241312475. doi: 10.1177/11786469241312475 (PMC11826855; doi:10.1177/11786469241312475)

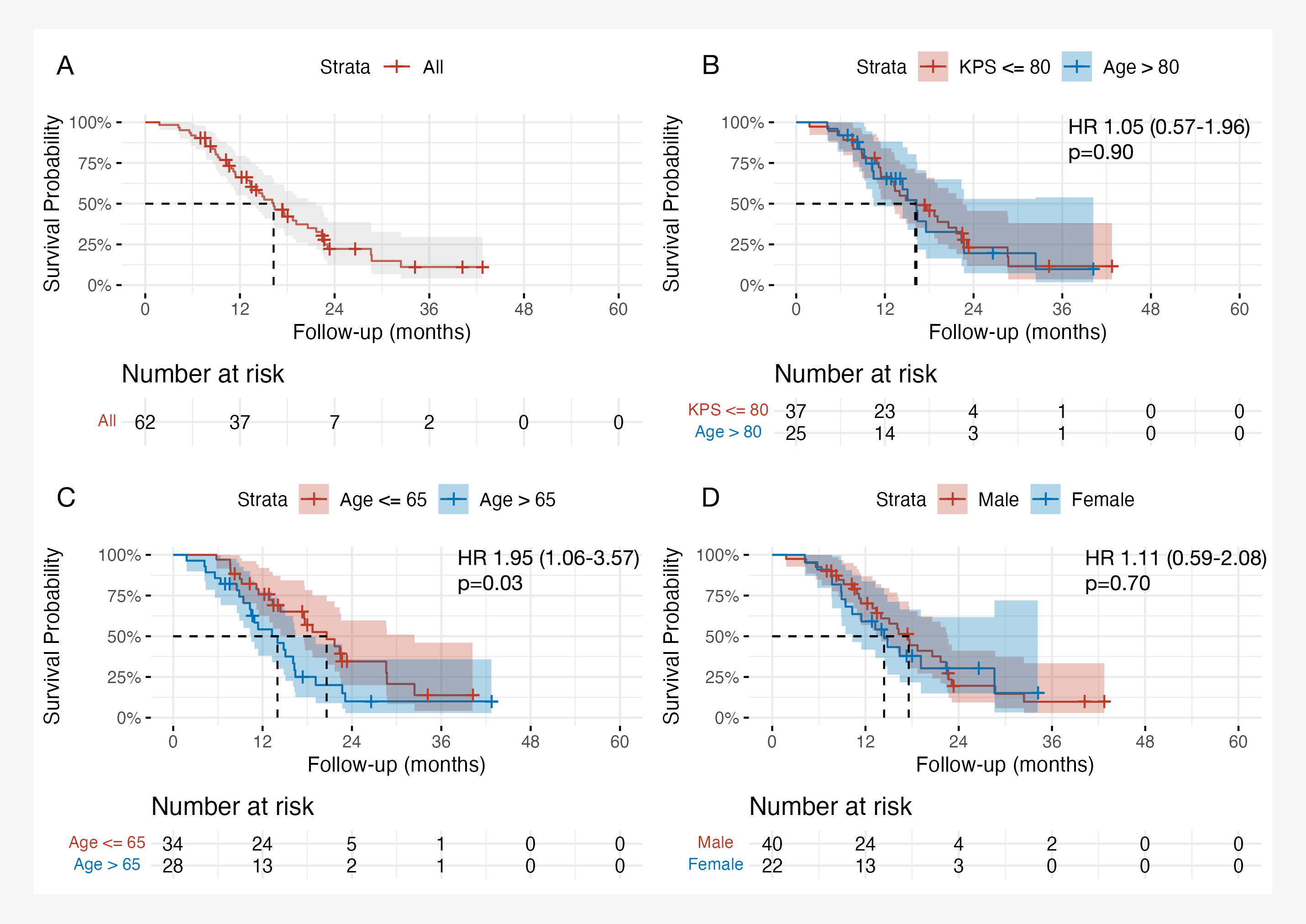

Supplement: sj-tif-1-try-10.1177_11786469241312475 – Supplemental material for Preoperative Peripheral Blood Serotonin and Kynurenine Levels Are Associated With Oncological Outcomes in Glioblastoma IDH-wt Patients [file sj-tif-1-try-10.1177_11786469241312475.tif]
